# The Quantitative Nanomechanical Mapping of Starch/Kaolin Film Surfaces by Peak Force AFM

**DOI:** 10.3390/polym13020244

**Published:** 2021-01-12

**Authors:** Anita Kwaśniewska, Michał Świetlicki, Adam Prószyński, Grzegorz Gładyszewski

**Affiliations:** Department of Applied Physics, Lublin University of Technology, 20-618 Lublin, Poland; m.swietlicki@pollub.pl (M.Ś.); a.proszynski@pollub.pl (A.P.); g.gladyszewski@pollub.pl (G.G.)

**Keywords:** AFM PeakForce QNM, nanomechanics, biopolymers, starch composite film, adhesion force

## Abstract

Starch films modified with additives are materials increasingly being used in the production of packaging. These types of biopolymers can, to a considerable degree, replace plastic, contributing to the reduction in both production and waste management costs. However, they should be characterised by specific mechanical and surface parameters which determine their application. In the presented work, the PeakForce Quantitative Nanomechanics Mapping (PFQNM) method was applied to analyse a starch-based biopolymer modified with two different kaolin clay contents (5% and 10%). The technique used facilitates the assessment of the correlation of Atomic Force Microscope AFM height parameters with nanomechanical ones which provide the definitions of mutual interactions and allow the possibility to analyse materials in respect of various details. The investigated material was mapped in the Derjaguin–Muller–Toporov (DMT) modulus, adhesion and height domains. The results obtained indicated the impact of additives on the determined parameters. Increases in the DMT modulus and the adhesion force, along with the kaolin content, were observed. The enhancement of starch films with kaolin clay also induced growth in the surface roughness parameters.

## 1. Introduction

Films based on biopolymers are becoming more developed due to sustainable development and environmental issues. Biodegradable materials produced from renewable and environmentally friendly resources have the capacity to replace synthetic products, for example, in the food packaging industry [1].

Notwithstanding the papers already published, intensive research is still being carried out to improve the quality and properties of biomaterials, including the possibility of using biomaterials films [2,3,4]. Therefore, biopolymers such as starch, chitosan and PLA are being blended with synthetic polymers [5,6,7,8], and biomatrices are being reinforced by fibres and particles [2,9,10,11]. Nanoadditives, such as layered silicates (e.g., bentonite [12], montmorillonite [13,14], wollastonite [15], kaolin [16,17] and talc [18]), carbon nanotubes [19,20], silver nanoparticles [14], cuttlebone powder [21] and essential oils [22,23], showed good dispersion and compatibility with biopolymer matrices. In this work, as a nanoadditive, kaolin clay was applied, which is widely regarded in industry as a thickener and substance carrier [24,25].

Packaging constitutes a protective barrier between the product and the environment. Therefore, testing the mechanical properties of packaging materials is a crucial benchmark assessment of their use. Elastic materials deform more easily, but have higher puncture resistance, while stiffer materials deform less, but have lower puncture resistance. The packaging also performs the product’s marketing and identification functions. Therefore, the surface properties are essential, affecting the aesthetic appearance and determining the possibility of printing or sticking information on them. The strength properties, and specific surface features, e.g., adhesion, can be directly determined using scanning probe microscopy.

PeakForce Quantitative Nanomechanics Mapping (PFQNM) is a relatively new AFM technique which facilitates the simultaneous mapping of surface morphology and mechanical parameters, such as adhesion, deformation, Young’s modulus and energy dissipation [26,27], with the same spatial resolution. PeakForce QNM allows the correlation of AFM surface morphology imaging (height parameters) with the quantitative mapping of nanomechanical parameters for the known surface areas. The correlation of AFM height parameters with nanomechanical parameters facilitates the detection of relationships and enables the analysis of materials from a different perspective.

The range of application of the PFQNM measurement, according to the Bruker Corporation, reaches the value of hundreds GPa when a diamond scanning probe [28] is used. To determine the Young modulus, two Hertzian mechanical models such as Johnson–Kendal–Roberts (JKR) and Derjaguin–Muller–Toporov (DMT) may be used. It is reported that the DMT model is typically applied to determine the Young’s modulus on the basis of PFQNM measurements, especially when a small tip-end radius is used [6,27,29,30].

The PFQNM is widely used to determine nanomechanical features and the interface of polymers, polymers blends and nanocomposite surfaces [31,32]. Materials with a low Young’s modulus value, such as water nanobubbles [33], biological materials (0.6 MPa) [26,34], polyurethanes (0.6 GPa) [35], starch/PVA blends (0.1–0.8 MPa) [6], PLA/PCL (1–4 GPa) [36], polystyrene (2.7 GPa) [35], PLA/PC (2–6 GPa) [37], toughened epoxy resin (3–6.2 GPa) [27], chitin/silica (30 GPa) [38] and bituminous materials [30], have mainly been studied. However, several pieces of research have also involved rigid materials, such as polished glass surfaces (78.8 GPa) [39], highly oriented pyrolytic graphite (HOPG) (31.1 GPa), fused silica (FS) (69.7 GPa), gold (179.8 GPa) and silicon (347.6 GPa) [40], for which the nanomechanical surface parameters were determined using the AFM quantitative method.

To the best of the authors’ knowledge, PFQNM measurement has so far not been used to determine the surface nanomechanical parameters of starch films with kaolin additions. Therefore, this study aimed to characterise the new biopolymer composites’ surface parameters using the PFQNM method, and to compare the obtained adhesive force values with the wettability described by the contact angles presented in an earlier publication [16].

## 2. Materials and Methods

### 2.1. Materials

Tested biopolymer films were prepared with raw potato starch (Melvit S.A., Warsaw, Poland) which had not been modified by any chemical, physical or enzymatic processes. Distilled water was used as a solvent for a polymer solution, and glycerol 99.5% (Avant Performance Materials S.A., Gliwice, Poland) was added as the plasticiser. As an additive, kaolin clay (China clay KOC from Valentine Clays Ltd., Stoke-on-Trent, UK) was used [16]. The biopolymer films were prepared by the widely used casting method [15,16]. The material preparation method enabled homogeneous materials to be obtained under controlled atmospheric conditions. Samples were prepared with 0%, 5% and 10% kaolin in relation to the starch mass, respectively. In the study, these samples were marked as k0, k5 and k10. It should be mentioned that the material preparation method procedure allowed homogeneous materials to be obtained under controlled atmospheric conditions.

### 2.2. AFM PeakForce QNM

**Figure 1:**
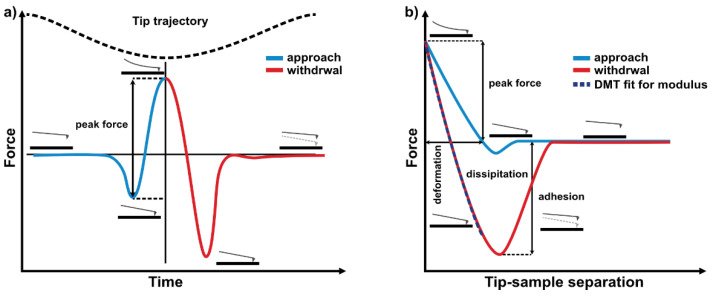
(**a**) Scheme of the force curves for a cantilever operating in Peak Force Tapping, (**b**) scheme of the single cycle force vs. separation curve with the dark blue dashed line fitted to the retract curve using the Derjaguin–Muller–Toporov (DMT) model.

Quantitative nanomechanical analysis of the samples was conducted using a MultiMode 8 with a NanoScope V controller equipped with a J-type scanner (Bruker, Billerica, MA, USA), using the PeakForce QNM mode. PFQNM is based on the Peak Force Tapping mode, wherein the material property mapping is based on the individual force vs. the separation curves obtained from each tap (Figure 1a) [41]. Each force curve represents a deflection of the probe lever relative to the change in the z-axis piezo position (Figure 1b). The shift in deflection is caused by differences in the interaction between the tip and the sample surface. The force curve is logged for each map pixel of the scanned image, which provides the mapping of all the investigated properties in a single scan line.

Each scan was performed with a 1 Hz rate and a scan size of 512 × 512 points, which is equivalent to a 262,144 force vs. separation curves. For each of the tested samples, scans of three noncontiguous areas of 10 × 10 μm were collected. Measurements were conducted at room temperature and 40% relative humidity. The z-range was set on 300 nm.

#### 2.2.1. AFM PFQNM Calibration

For quantitative AFM measurements, it is necessary to employ a calibration process. For this study, the so-called absolute method was used. This method consists of the direct measurement of the cantilever tip-end radius and the spring constant. The main advantage of the applied approach is that the standard sample with a known modulus close to the measured sample is not required. The first step is to calibrate the deflection sensitivity on the sapphire sample which quantifies the photodetector response. The exact spring constant of the cantilever is determined by the Thermal Tune function. The tip radius is measured by a standard titanium sample using the Tip Qualification function on NanoScope software (Bruker, Billerica, MA, USA). After completing the calibration process, the measurement parameters in the PFQNM mode are verified on a calibration sample with known mechanical parameters.

A Vtespa 300 probe with a nominal spring constant of 42 N/m, a nominal tip radius of 5 nm, a length of 140 µm and a width of 38 µm was used in the study. The probe was suitable for the expected range of modulus values [28]. The values of the spring constant and the tip radius determined in the calibration process were 44 N/m and 17 nm, respectively.

#### 2.2.2. DMT Modulus

The Young’s modulus was calculated by fitting part of the unloading curve using the Derjaguin–Muller–Toporov (DMT) model of elastic interaction due to consideration of the adhesion force [28]. The fitting region was set between 90% and 30% of the force range for the retract curve. This model is suitable for testing stiff materials characterised by low adhesion values using a probe with a small tip radius.

The reduced Young’s modulus E* was obtained by fitting the retract curve to the DMT model [42]:

(1) $$F-F_{adh}=\frac{4}{3}E^{*}\sqrt{R{\left(d-d_{0}\right)}^{3}}$$

where *F − F_adh_* is the force on the cantilever relative to the adhesion force, *R* is the tip-end radius, and (*d* – *d*_0_) is the deformation of the sample. Therefore, the standard value of sample Poisson ratio 0.3 was used, and it was assumed that the tip modulus was infinite. This procedure allowed the sample modulus to be calculated [28] as follows:

(2) $$E^{*}={\left[\frac{1-\nu_{s}^{2}}{E_{s}}+\frac{1-\nu_{tip}^{2}}{E_{tip}}\right]}^{-1}$$

where *E_tip_* is the tip modulus, *E_s_* the sample modulus, *ν_tip_* the tip Poisson ratio, and *ν_s_* the sample Poisson ratio.

The calculations of the modulus with the DMT model were taken in real time during the scan for every probe tap.

#### 2.2.3. Adhesion Force

The adhesion force was determined as the lowest point in the force vs. distance curves, as shown in Figure 1b. It should be mentioned that the process of tip functionalisation was not performed, as all the measurements were based on the tip parameters calibration.

### 2.3. Statistical Analysis

Statistical analyses, including histograms of the data obtained from the measurements, were performed with the software package Statistica 13.1 (TIBCO Software Inc., Palo Alto, CA, USA). Due to the lack of normal distribution in the analysed data, Kruskal–Wallis ANOVA with multiple comparisons of mean ranks was used to assess the differences between the experimental groups. The significance level *p* was set at 0.05.

## 3. Results and Discussion

**Figure 2:**
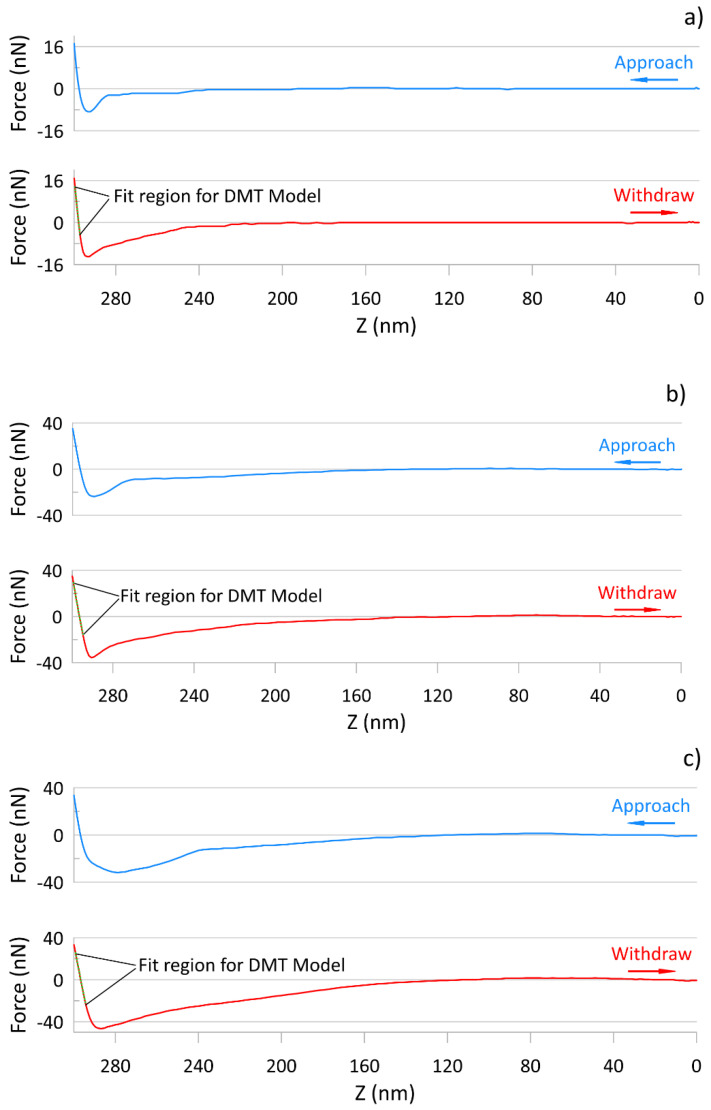
Typical force curves for samples k0 (**a**), k5 (**b**), k10 (**c**), with marked fitting regions for the DMT model.

The results obtained are presented in the form of maps of height, DMT modulus and adhesion force. The distribution of the measured parameters is shown in the histograms. Histograms of the DMT modulus and adhesion force are based on every single point of the measured 512 × 512 scans, therefore in all cases, the number of counts was 262,144. Data from three noncontiguous areas for each of the tested samples were used for analysis. Sample force curves obtained for k0, k5 and k10 films are presented in Figure 2.

**Figure 3:**
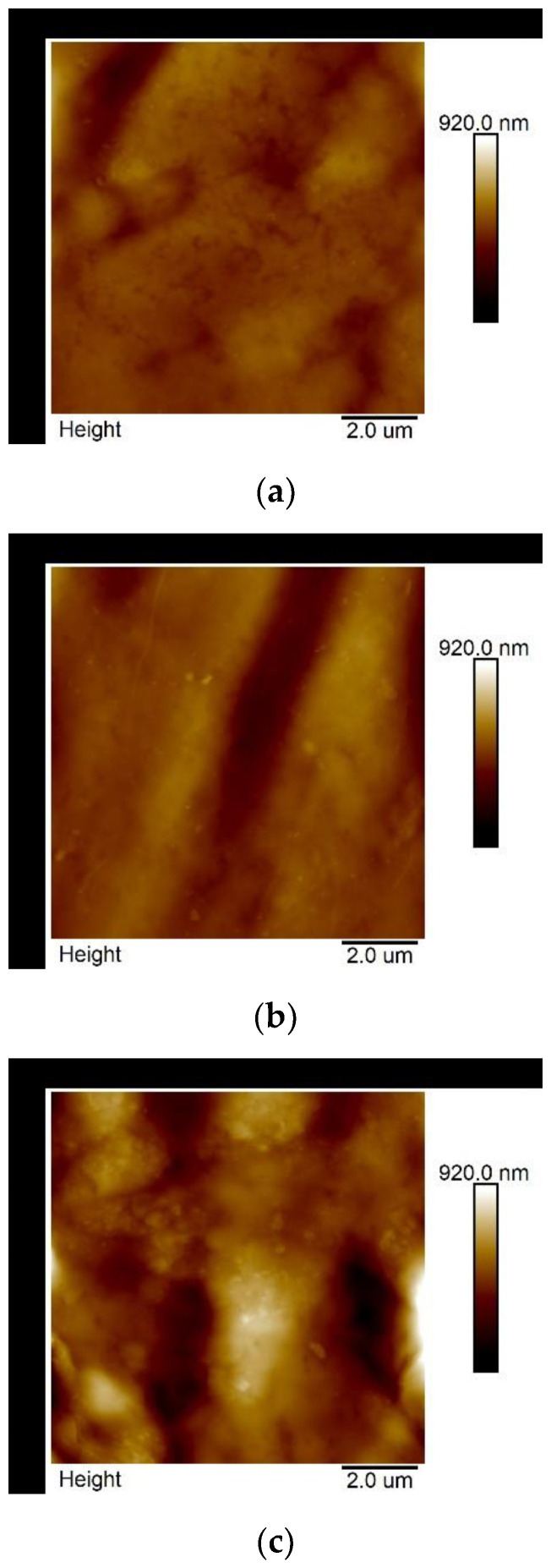
The representative map of topography (10 × 10 μm) for samples k0 (**a**), k5 (**b**), k10 (**c**).

**Figure 4:**
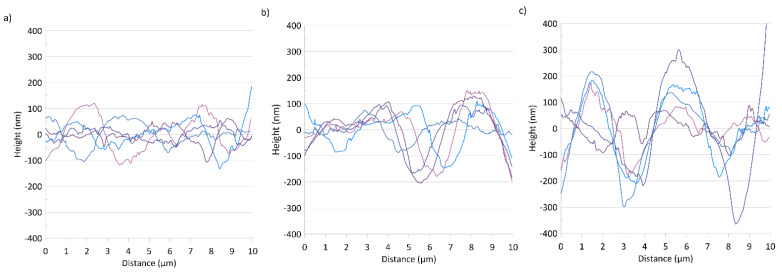
Five representative cross-sections obtained from evenly distributed areas over the entire surface of the films for samples k0 (**a**), k5 (**b**) and k10 (**c**).

The representative 2D maps of surface topography in the height domain are presented in Figure 3. The images were corrected to eliminate irregularities associated with the shape of the samples (3rd order plane fit). The surface morphology changed with the increase in nanoadditive content in the biopolymer matrix, as shown in Figure 3. The exemplary cross-sections (Figure 4a–c) present the rise in the height value, probably caused by a more disordered biopolymer structure resulting from the intercalation of the kaolin particles, especially in the case of sample k10.

The root mean square heights (S_q_) and the arithmetical mean heights (S_a_) of the surfaces were determined by NanoScope 1.9 software. The surface roughness parameters increased with increases in the concentration of the nanoclay in the biopolymer matrix. The S_a_ and S_q_ values for k0 were 32.03 and 41.8 nm; for k5, 41.3 and 56.3 nm; and for k10, 77.2 and 100.8 nm, respectively, which is also visible on the height profiles (Figure 4). A similar effect was obtained by Świetlicka et al. [43] for starch films modified by wollastonite.

Along with the changes observed in the surface topography (Figure 3 and Figure 4), caused by increases in the additive content, changes in the DMT modulus and adhesion force values were also noted.

The statistical analysis (Table 1) showed that the DMT moduli of the samples were significantly different, while, when adhesion force was concerned, noticeable differences were only visible between the k0 and k10 composites.

**Figure 5:**
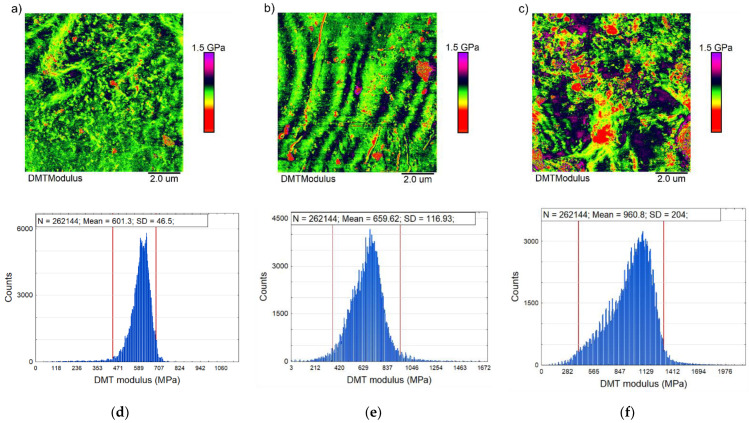
Representative map of the DMT moduli (10 × 10 μm) for samples k0 (**a**), k5 (**b**), k10 (**c**), and the corresponding histogram (**d**–**f**) of the distribution of the DMT moduli. The red line superimposed on the frequency histograms represents the 95% range of the measured values.

Figure 5 shows the obtained DMT modulus maps. As can be seen, the k0 film surface is characterised by a uniform value of the Young’s modulus over the entire tested area, which is proven by the small dispersion of values on the histogram of the DMT modulus (Figure 5d) over the range 456.6 to 780 MPa. A similar effect was observed by Panitescu et al. [6] for starch/PVA.

The 5% kaolin addition to the biopolymer matrix led to an increase in the dispersion of the Young’s modulus values from 505.7 to 1120 MPa, as shown in Figure 5b and in the corresponding histogram (Figure 5e). A further increase in the additive for the k10 film caused an increase in the mean value of the Young’s modulus, as well as the distribution range from 439 to 1926.6 MPa (Figure 5f).

The histograms of modulus distribution for nanocomposites k5 and k10 showed no splitting areas, which would have suggested a clear phase separation between the kaolin and the matrix. The applied nanoadditives were homogeneously combined with the biopolymer and changed the average values of the modules. The observed increase in the DMT modulus values for the k5 and k10 nanocomposite films indicates altered starch matrix chain mobility as compared to the k0 film. This was caused by a hydrogen bond of the kaolin clay with a biopolymer, which reduced the mutual movement of the polymer chains, as was confirmed by Chivrac et al. [44] for starch nanobiocomposites.

**Figure 6:**
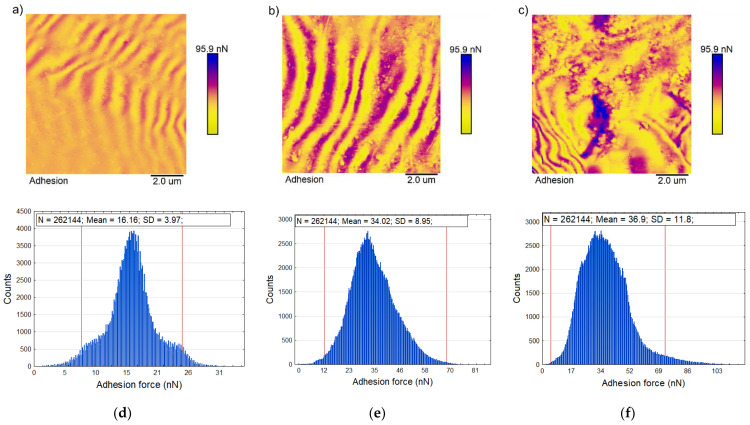
Representative map of the adhesion forces (10 × 10 μm) for samples k0 (**a**), k5 (**b**), k10 (**c**), and the corresponding histogram (**d**–**f**) of the distribution of the adhesion forces. The red line superimposed on the frequency histograms represents the 95% range of the measured values.

Similar to the DMT modulus value for the k0 sample, adhesion forces are relatively homogeneous over the whole scan area. The histogram of adhesion distribution shows the scatter of the obtained values ranging from 6.9 to 21.5 nN, with an average value of 14.2 nN (Table 1). The addition of kaolin increased the values of the mean adhesive forces for samples k5 and k10 up to 34.5 and 42.6 nN, respectively, as seen in Figure 2. The corresponding histograms (Figure 6d–f) show an increase in the section for the measured values of the adhesion forces, which reached values ranging from 15.9 to 53.7 nN, and from 18.3 to 75.6 nN, respectively.

The adhesion force distribution for the k5 and k10 composites (Figure 6d–f) shows no peak splitting phenomenon, which indicates no separation of the additions or the polymer phase. This confirms good compatibility between the starch matrix and the kaolin nanofillers, which blend with the matrix homogeneously without feature agglomerations. The histograms (Figure 6d–f) show that kaolin, by combining with the starch matrix, formed interfacial interaction areas with good biopolymer/kaolin interface adhesion. Their amounts in the nanocomposite increased with increases in nanofiller content.

Comparing the DMT modulus and the adhesion force maps made it possible to observe that the areas of higher modulus and adhesion force values are correlated. The increasing amount of kaolin addition in the starch biopolymer matrix resulted in an increase in Young’s modulus and the limited mobility of the polymer chains. Furthermore, kaolin in the polymer matrix maintained a positively charged surface, influencing probe deformation during the measurement. This effect increased with increasing kaolin content and can be seen on the force curves for k5 and k10 (Figure 2), which show an increased range of influence on the tip. This effect is probably caused by the attractive forces occurring between the positive charges of the kaolin surface and the tip ends.

The obtained values of the adhesion forces registered between the tip and the sample surface can be compared with the wettability of the film surfaces. In an earlier work, the authors determined the wettability of the films by measurement of the contact angles [16]. The wettability of the surface films was assessed by the static sessile drop method. The contact angle was determined based on the geometry of the water drops on the tested surfaces. The results obtained are also included in Table 1.

The relation between contact angle and adhesion force indicates that an increase in the adhesion force is associated with a decrease in the contact angle. The k5 and k10 nanocomposite films’ wettability increased, resulting in more hydrophilic surfaces. Krotil et al. and Vlassov et al. reported that a silicon tip showed higher adhesive forces with a hydrophilic surface than with a hydrophobic surface, therefore, changes in hydrophilicity resulted in changes in the adhesion values [45,46]. Laitinen et al. reported that the interaction forces between the tip and the sample surface depended on surface geometry, and indicated that the value of adhesion increased with increasing surface roughness, which our results confirm [47].

## 4. Conclusions

In this study, the PeakForce Quantitative Nanomechanical Mapping (PFQNM) mode was used for the simultaneous mapping of the quantitative nanomechanical surface parameters and topography of starch/kaolin films with a high resolution.

The samples were prepared with 0%, 5% and 10% kaolin in relation to starch mass, respectively. With the increase in kaolin addition, the average value of the DMT moduli and the values of the mean adhesive forces increased. The composite material with a higher modulus value became stiffer, thus it could endure more load and underwent less deformation.

Because the higher modulus values and adhesion force areas obtained on the maps correspond to each other, we can assume that attractive forces occurred between the kaolin surface and the end of the tip. Additionally, the obtained values of the adhesion forces between the tip and the sample surface were compared with the wettability of the film surfaces, which indicated that an increase in the adhesion force is associated with a decrease in the contact angle. The k5 and k10 nanocomposite films’ wettability increased, resulting in more hydrophilic surfaces.

To conclude, the applied PFQNM method proved to be an appropriate tool for the determination of the mechanical parameters of biopolymer surfaces and allowed for the evaluation of the surface features reflecting their structures.

## Figures and Tables

**Table 1:** The mean values of the measured surface parameters for 3 areas for each sample.

Sample	DMT Modulus, (MPa)	Adhesion Force, (nN)	Contact Angle, (°)
k0	598.54 ± 65.12 ^a^	14.18 ± 3.0 ^a^	92.69 ± 8.82 ^a^
k5	757.60 ± 115.25 ^b^	34.5 ± 8.53 ^ab^	48.03 ± 12.1 ^b^
k10	1018.71 ± 297.21 ^c^	42.57 ± 11.92 ^b^	46.66 ± 11.58 ^b^

^a–c^ Superscripts in the same column mark statistically significantly different groups at *p* < 0.05.

## References

[1] Youssef A.M., El-Sayed S.M. (2018). Bionanocomposites materials for food packaging applications: Concepts and future outlook. Carbohydr. Polym..

[2] John M.J., Thomas S. (2008). Biofibres and biocomposites. Carbohydr. Polym..

[3] Averous L., Boquillon N. (2004). Biocomposites based on plasticized starch: Thermal and mechanical behaviours. Carbohydr. Polym..

[4] Ponce A., Roura S.I., Moreira M.R. (2016). Casein and Chitosan Polymers: Use in Antimicrobial Packaging. Antimicrobial Food Packaging.

[5] Arboleda G.A., Montilla C.E., Villada H.S., Varona G.A. (2015). Obtaining a Flexible Film Elaborated from Cassava Thermoplastic Starch and Polylactic Acid. Int. J. Polym. Sci..

[6] Panaitescu D.M., Frone A.N., Ghiurea M., Chiulan I. (2015). Influence of storage conditions on starch/PVA films containing cellu-lose nanofibers. Ind. Crops Prod..

[7] Aydemir D., Gardner D.J. (2020). Biopolymer blends of polyhydroxybutyrate and polylactic acid reinforced with cellulose nano-fibrils. Carbohydr. Polym..

[8] Bonilla J., Fortunati E., Vargas M., Chiralt A., Kenny J.M. (2013). Effects of chitosan on the physicochemical and antimicrobial properties of PLA films. J. Food Eng..

[9] Ma X., Yu J., Kennedy J.F. (2005). Studies on the properties of natural fibers-reinforced thermoplastic starch composites. Carbohydr. Polym..

[10] Wu Q., Zhang P., Zhang Y., Fan B., Zhu M., Wu Z. (2011). Thermoplastic starch modified with hydrophobic polyurethane microparticles. Starch Stärke.

[11] Vigneshwaran N., Ammayappan L., Huang Q. (2011). Effect of Gum arabic on distribution behavior of nanocellulose fillers in starch film. Appl. Nanosci..

[12] Erba S.Ç., Baştürk S.B. (2019). Fabrication and Characterization of Nanoclay-Reinforced Thermoplastic Composite Films. Mater. Tehnol..

[13] Cyras V.P., Manfredi L.B., Ton-That M.T., Vázquez A. (2008). Physical and mechanical properties of thermoplastic starch/montmorillonite nanocomposite films. Carbohydr. Polym..

[14] Cheviron P., Gouanvé F., Espuche E. (2016). Preparation, characterization and barrier properties of silver/montmorillonite/starch nanocomposite films. J. Membr. Sci..

[15] Kwasniewska A., Muszynski S., Tatarczak J., Gładyszewski G., Gładyszewska B. (2016). Wollastonite-filled and Arabic gum-modified starch films. Part 1. Mechanical and structural properties. Przem. Chem..

[16] Kwaśniewska A., Chocyk D., Gładyszewski G., Borc J., Świetlicki M., Gładyszewska B. (2020). The Influence of Kaolin Clay on the Mechanical Properties and Structure of Thermoplastic Starch Films. Polymers.

[17] Mbey J.A., Hoppe S., Thomas F. (2012). Cassava starch-kaolinite composite film. Effect of clay content and clay modification on film properties. Carbohydr. Polym..

[18] Świetlicki M., Chocyk D., Klepka T., Prószyński A., Kwaśniewska A., Borc J., Gładyszewski G. (2020). The structure and mechanical properties of the surface layer of polypropylene polymers with talc additions. Materials.

[19] Jose J., De S.K., Ma’adeed M.A.A., Dakua J.B., Sreekumar P.A., Sougrat R., Al-Harthi M.A. (2015). Compatibilizing role of carbon nanotubes in poly(vinyl alcohol)/starch blend. Starch Stärke.

[20] Yurdakul H., Durukan O., Seyhan A.T., Celebi H., Oksuzoglu M., Turan S. (2013). Microstructural characterization of corn starch-based porous thermoplastic composites filled with multiwalled carbon nanotubes. J. Appl. Polym. Sci..

[21] Bootklad M., Kaewtatip K. (2015). Biodegradability, mechanical, and thermal properties of thermoplastic starch/cuttlebone composites. Polym. Polym. Compos..

[22] Campos-Requena V.H., Rivas B.L., Pérez M.A., Figueroa C.R., Figueroa N.E., Sanfuentes E.A. (2017). Thermoplastic starch/clay nanocomposites loaded with essential oil constituents as packaging for strawberries—In vivo antimicrobial synergy over Bo-trytis cinerea. Postharvest Biol. Technol..

[23] Souza A.C., Goto G.E.O., Mainardi J.A., Coelho A.C.V., Tadini C.C. (2013). Cassava starch composite films incorporated with cinnamon essential oil: Antimicrobial activity, microstructure, mechanical and barrier properties. LWT Food Sci. Technol..

[24] Muszyński S., Kwaśniewska A., Sołowiej B., Tomczyk A., Leus A., Szymanek M., Siedliska K., Gladyszewska B. (2017). Physical properties of kaolin clay-containing pectin gels. Właściwości fizyczne żeli pektynowych zawierających glinkę kaolinową. Przem. Chem..

[25] Zhang B., Chang Z., Li J., Li X., Kan Y., Gao Z. (2019). Effect of kaolin content on the performances of kaolin-hybridized soybean meal-based adhesives for wood composites. Compos. Part B Eng..

[26] Adamcik J., Berquand A., Mezzenga R. (2011). Single-step direct measurement of amyloid fibrils stiffness by peak force quantitative nanomechanical atomic force microscopy. Appl. Phys. Lett..

[27] Moosburger-Will J., Jäger J., Horn S., Wellhausen C. (2012). Investigation of phase morphology of polyetherimide-toughened epoxy resin by scanning probe microscopy. Polym. Test..

[28] Pittenger B., Erina N., Su C. (2010). Application Note—Quantitative Mechanical Property Mapping at the Nanoscale with PeakForce QNM..

[29] Young T.J., Monclus M.A., Burnett T.L., Broughton W.R., Ogin S.L., Smith P.A. (2011). The use of the PeakForceTM quantitative nanomechanical mapping AFM-based method for high-resolution Young’s modulus measurement of polymers. Meas. Sci. Technol..

[30] Nahar S.N., Schmets A.J.M., Schitter G., Scarpas A. Quantitative nanomechanical property mapping of bitumen micro-phases by peak-force Atomic Force Microscopy. Proceedings of the International Conference Asphalt Pavements, ISAP 2014.

[31] Banerjee S.S., Kumar K.D., Sikder A.K., Bhowmick A.K. (2015). Nanomechanics and Origin of Rubber Elasticity of Novel Nanostructured Thermoplastic Elastomeric Blends Using Atomic Force Microscopy. Macromol. Chem. Phys..

[32] Ren M., Shi T., Corr D.J., Shah S.P. (2019). Mechanical Properties of Micro-regions in Cement-based Material based on the Peak-Force QNM Mode of AFM. J. Wuhan Univ. Technol. Mater. Sci. Ed..

[33] Zhao B., Song Y., Wang S., Dai B., Zhang L., Dong Y., Lü J., Hu J. (2013). Mechanical mapping of nanobubbles by PeakForce atomic force microscopy. Soft Matter.

[34] Smolyakov G., Formosa-Dague C., Severac C., Duval R.E., Dague E. (2016). High speed indentation measures by FV, QI and QNM introduce a new understanding of bionanomechanical experiments. Micron.

[35] Dokukin M.E., Sokolov I. (2012). Quantitative Mapping of the Elastic Modulus of Soft Materials with HarmoniX and PeakForce QNM AFM Modes. Langmuir.

[36] Zhang S., Liu H., Gou J., Ying J., Wang Y., Liu C., Shen C. (2019). Quantitative nanomechanical mapping on poly(lactic ac-id)/poly(ε-caprolactone)/carbon nanotubes bionanocomposites using atomic force microscopy. Polym. Test..

[37] Qu Z., Bu J., Pan X., Hu X. (2018). Probing the nanomechanical properties of PLA/PC blends compatibilized with compatibilizer and nucleation agent by AFM. J. Polym. Res..

[38] Smolyakov G., Pruvost S., Cardoso L., Alonso B., Belamie E., Duchet-Rumeau J. (2016). AFM PeakForce QNM mode: Evidencing nanometre-scale mechanical properties of chitin-silica hybrid nanocomposites. Carbohydr. Polym..

[39] Hopf J., Pierce E.M. (2014). Topography and Mechanical Property Mapping of International Simple Glass Surfaces with Atomic Force Microscopy. Procedia Mater. Sci..

[40] Zeng G., Dirscherl K., Garnæs J. (2018). Toward Accurate Quantitative Elasticity Mapping of Rigid Nanomaterials by Atomic Force Microscopy: Effect of Acquisition Frequency, Loading Force, and Tip Geometry. Nanomaterials.

[41] Xu K., Sun W., Shao Y., Wei F., Zhang X., Wang W., Li P. (2018). Recent development of PeakForce Tapping mode atomic force microscopy and its applications on nanoscience. Nanotechnol. Rev..

[42] Derjaguin B.V., Muller V.M., Toporov Y.P. (1975). Effect of contact deformations on the adhesion of particles. J. Colloid Interface Sci..

[43] Świetlicka I., Muszyński S., Kwaśniewska A., Świetlicki M., Gołacki K., Gładyszewska B. (2017). Wollastonite-filled and arabic gum-modified starch films. Part 4. Surface nanostructure. Folie skrobiowe napełniane wollastonitem i modyfikowane gumaą arabskaą. Cz. IV. Nanostruktura powierzchni. Przem. Chem..

[44] Chivrac F., Pollet E., Schmutz M., Avérous L. (2010). Starch nano-biocomposites based on needle-like sepiolite clays. Carbohydr. Polym..

[45] Krotil H.U., Stifter T., Waschipky H., Weishaupt K., Hild S., Marti O. (1999). Pulsed force mode: A new method for the investiga-tion of surface properties. Surf. Interface Anal..

[46] Vlassov S., Oras S., Antsov M., Sosnin I., Polyakov B., Shutka A., Dorogin L.M. (2018). Adhesion and Mechanical Properties of PDMS-Based Materials Probed with AFM: A Review. Rev. Adv. Mater. Sci..

[47] Laitinen O., Bauer K., Niinimäki J., Peuker U.A. (2013). Validity of the Rumpf and the Rabinovich adhesion force models for alu-mina substrates with nanoscale roughness. Powder Technol..

